# Active Music Engagement and Cortisol as an Acute Stress Biomarker in Young Hematopoietic Stem Cell Transplant Patients and Caregivers: Results of a Single Case Design Pilot Study

**DOI:** 10.3389/fpsyg.2020.587871

**Published:** 2020-11-02

**Authors:** Steven J. Holochwost, Sheri L. Robb, Amanda K. Henley, Kristin Stegenga, Susan M. Perkins, Kristen A. Russ, Seethal A. Jacob, David Delgado, Joan E. Haase, Caitlin M. Krater

**Affiliations:** ^1^WolfBrown, Cambridge, MA, United States; ^2^School of Nursing, Indiana University, Indianapolis, IN, United States; ^3^Children’s Mercy Hospital, Kansas City, MO, United States; ^4^School of Medicine, Indiana University, Indianapolis, IN, United States; ^5^Richard M. Fairbanks School of Public Health, Indiana University, Indianapolis, IN, United States; ^6^Melvin and Bren Simon Comprehensive Cancer Center, Indiana University, Indianapolis, IN, United States; ^7^Riley Hospital for Children at Indiana University Health, Indianapolis, IN, United States; ^8^Astellas Pharma Global Development, Northbrook, IL, United States

**Keywords:** music therapy, HPA axis (hypothalamus-pituitary-adrenal), cortisol, hematopoietic (stem) cell transplantation (HCT), HSCT

## Abstract

This paper reports the results of a single case design pilot study of a music therapy intervention [the Active Music Engagement (AME)] for young children (age 3.51 to 4.53 years) undergoing hematopoietic stem cell transplantation (HCST) and their caregivers. The primary aims of the study were to determine feasibility/acceptability of the AME intervention protocol and data collection in the context of HCST. Secondary aims were to examine caregivers’ perceptions of the benefit of AME and whether there were changes in child and caregiver cortisol levels relative to the AME intervention. Results indicated that the AME could be implemented in this context and that data could be collected, though the collection of salivary cortisol may constitute an additional burden for families. Nevertheless, data that were collected suggest that families derive benefit from the AME, which underscores the need for devising innovative methods to understand the neurophysiological impacts of the AME.

## Introduction

Hematopoietic stem cell transplantation (HSCT) is used to treat malignant conditions that require high dose chemotherapy (and at times radiation therapy), as well as non-malignant conditions such as hemoglobinopathies (Guilcher et al., 2018). HSCT is a high-intensity, complex treatment that has many risks, with many pediatric patients and their caregivers experiencing high levels of emotional distress during transplant. Compared with other age groups, young children undergoing HSCT and their caregivers are at particular risk for heightened emotional distress, which is associated with physical symptom distress, as well as diminished quality of life and family function (Kazak et al., 1997, 2005; Best et al., 2001; Santacroce, 2002; Kazak and Baxt, 2007; Ingerski et al., 2010; Graf et al., 2013; Virtue et al., 2014). In addition, this acute emotional distress is related to traumatic stress symptoms after treatment ends (Stuber et al., 1996, 1997; Kazak et al., 1998; Hobbie et al., 2000; Kangas et al., 2002; Bruce, 2006; Ingerski et al., 2010). To date, few interventions have been developed to address the interrelated distress experienced by young children and their caregivers during acute medical treatment (Hatfield et al., 1993; Stuber et al., 1996; Kazak et al., 1997; Robinson et al., 2007; Ingerski et al., 2010; Virtue et al., 2014), with even fewer interventions specific to HSCT (Robb and Hanson-Abromeit, 2014).

The active music engagement (AME) intervention uses music play experiences to diminish stressful qualities of the treatment environment, encouraging the use of positive coping strategies to reduce the emotional and traumatic distress experienced by young children (age 3–8 years) and their caregivers (Robb, 2000; Robb et al., 2008, 2017). Early studies established AME as beneficial in managing child emotional distress (Robb, 2000; Robb et al., 2008, 2017) and explored caregiver benefit (Robb et al., 2017); with an ongoing mechanistic trial aimed at identifying behavioral, sociological, and psychological variables responsible for change (NR015789). To date, all of the AME studies have focused on short inpatient admissions for chemotherapy and psychosocial mechanisms of action. However, given the intensity and length of HSCT, we anticipate that AME may have even greater benefit and clinical utility in this patient population where the average inpatient stay is 3–8 weeks. We anticipated that translation of AME for HSCT would likely require changes to the intervention protocol, followed by formal exploration concerning AME feasibility/acceptability during transplant. And, in order to expand our understanding about how active music interventions work to mitigate transplant-related stress, we decided to also explore the feasibility/acceptability of collecting cortisol (a stress biomarker) from children and caregivers.

**FIGURE 1:**
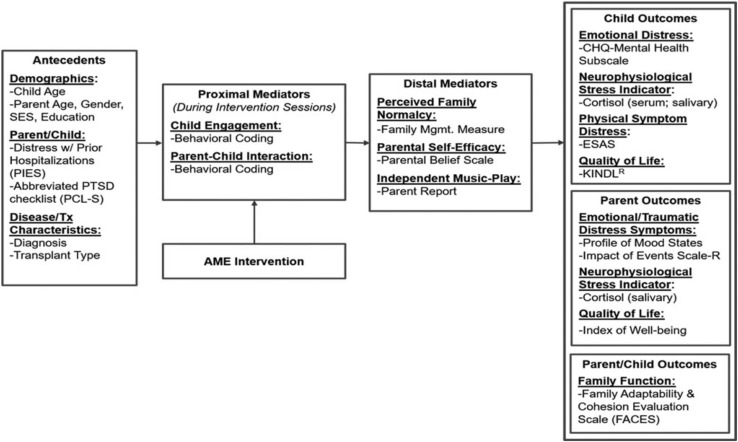
Conceptual framework. Reprinted from Russ et al. (2020).

The conceptual framework for this study is based on Robb’s Contextual Support Model of Music Therapy (Robb, 2000, 2003a,b) and further informed by Kazak’s Pediatric Medical Traumatic Stress Model (Kazak and Baxt, 2007; see Figure 1). In this framework, recurring events related to HSCT treatment (e.g., symptom distress; invasive procedures) are viewed as potentially traumatic. Pre-existing factors (i.e., antecedents) influence caregiver appraisal of whether an event is experienced as traumatic or not traumatic. Research indicates that higher child and caregiver distress during HSCT is related to demographics (child/caregiver age, socioeconomic status) (Kazak et al., 2003; Stevens et al., 2006), higher distress and greater traumatic stress symptom during prior hospitalizations (Kazak and Barakat, 1997; Best et al., 2001; Barrera et al., 2004), and disease and treatment characteristics (diagnosis/transplant type) (Stuber et al., 1997; Kazak et al., 1998; Hobbie et al., 2000; Langeveld et al., 2004). As illustrated in Figure 1, the AME is designed to directly target potential proximal mediators of child engagement and caregiver-child interaction (Robb, 2000; Robb et al., 2017), and distal mediators of perceived family normalcy (Knafl and Deatrick, 2002), caregiver confidence (self-efficacy) about their ability to support their child during treatment (Steele et al., 2003), and occurrence of independent music play between music therapist led sessions. Outcomes specified in the model include child outcomes (emotional distress, physical symptom distress, cortisol, and quality of life), caregiver outcomes (emotional distress, traumatic stress symptoms, cortisol, and quality of life), and family function.

Investigation of biological pathways underlying the use of music to mitigate transplant-related stress is supported by evidence that increased hypothalamic-pituitary-adrenal (HPA) axis activity stimulates the release and production of inflammatory biomarkers, which in turn is associated with negative health outcomes for people undergoing treatment for chronic health conditions and their caregivers (Padgett and Glaser, 2003; Glaser and Kiecolt-Glaser, 2005; Costanzo et al., 2011). Cortisol is a steroid hormone secreted by the HPA axis in response to acute and prolonged stress, and assaying bodily fluids (such as blood or saliva) for cortisol is a method commonly used to index HPA axis activity in children (Gunnar and Quevedo, 2007). Few intervention studies have looked at cortisol in pediatric patients with malignant or non-malignant conditions (Lane, 1991; Walco et al., 2005; Post-White et al., 2009), and of those only one used music (Lane, 1991) and none involved caregivers. This is likely due to challenges associated with cortisol collection and its interpretation during cancer and HSCT treatment. Equally challenging is the ability to conduct fully powered, randomized intervention trials in low-incidence populations [such is the case with pediatric cancer and sickle cell disease (SCD)] (Moore, 2004; Kang et al., 2010; Rensen et al., 2017). To overcome these challenges, we used a single-case design study, which allows for estimation of intervention effects in small samples that are drawn from low-incidence populations. Details of the study protocol were published by Russ et al. (2020), with outcomes reported in this manuscript.

### Current Study

As specified in our previously published protocol, the current study comprised two sets of aims (Russ et al., 2020). The primary aims were to: determine feasibility/acceptability of the AME intervention protocol during HSCT; evaluate clinical feasibility/acceptability of collecting data in this context, including collection of biological samples according to a rigorous schedule required when using a single-case design; and assess caregivers’ perspectives about the collection of these samples. Secondary aims were to examine caregivers’ perceptions of the benefit of AME and whether there were changes in child and caregiver cortisol levels relative to the AME intervention.

These aims generated five specific research questions:

(1) What proportion of eligible caregiver-child dyads consent to study participation, and what percentage of AME sessions do these dyads complete?
(2) What self-report and biological data can be collected during HSCT?
(3) What are caregivers’ perspectives about the relative ease/burden of biological sample collection?
(4) What are caregivers’ perceptions of the benefit (or non-benefit) of AME for managing distress, and enhancing family and quality of life outcomes for self and child?
(5) Are there observed changes in caregiver and/or child cortisol levels associated with the AME intervention?

## Materials and Methods

### Participants

To address these questions data were collected in a single-group pilot study approved by the Indiana University Institutional Review Board. To be eligible for study inclusion, children had to be no younger than 3 years of age and no older than 8, were required to be undergoing inpatient HSCT for a malignant or non-malignant condition, and could not have a significant cognitive impairment that might limit their participation. A consistent caregiver had to agree to be present for music therapy and data collection sessions, and both the participating caregiver and child had to speak English. Dyads with children undergoing autologous transplants – in which children’s own stem cells are used – and allogenic transplants, in which stem cells are provided by a donor, were included in the study.

Four child-caregiver dyads participated in the study. In each case, the caregiver was the child’s mother, three of whom identified as non-Hispanic white and one of whom identified as non-Hispanic black. Mothers ranged from 34 to 42 years of age, whereas children ranged from 3.51 to 4.53 years of age at the time their participation in the study began. Two children were identified by their mothers as non-Hispanic white, one as non-Hispanic black, and one as non-Hispanic, more than one race. Although approximately 5% of families with children in the city in which the study was conducted speak a language other than English as their first language, all participants in the study reported that English was their first language.

Children from two of the dyads were undergoing an autologous transplant, and two children were undergoing an allogenic transplant. In Table 1, we provide abbreviated information for each parent/child dyad (e.g., child age, gender, and transplant type) to assist with data interpretation, while protecting participant anonymity.

**TABLE 1:** Child demographics, diagnosis, and treatment.

**Participant number**	**Child age**	**Diagnosis**	**Transplant type**	**Conditioning agent**	**Acute GVHD**
1	4.53	Neuroblastoma	Autologous	Chemo only	No
2	3.81	Neuroblastoma	Autologous	Chemo only	No
3	3.51	Relapsed ALL	Allogeneic	Chemo only	Yes Stage 4, Grade III
4	3.51	Sickle cell disease	Allogeneic	Chemo only	No

*Age corresponds to the child’s age at the beginning of their participation in the study. GVHD, graft versus host disease.*

### Procedures

#### AME Therapy Sessions

All study participants were scheduled to receive two 45-min AME sessions each week that they were hospitalized for HSCT. All participants, regardless of transplant type, were expected to receive a minimum of eight AME sessions over 4-weeks. Each session was delivered to caregiver-child dyads in a private room by a board-certified music therapist (MT-BC) with 8 years’ experience working with children in acute hospital settings. The MT-BC tailored music-based play experiences to encourage active engagement in and independent use of music play as a way to manage distress. During sessions, the MT-BC provided repeated opportunities for caregivers and children to experience competence, autonomy, and meaningful interactions.

The AME intervention had three primary components: (1) therapist-led music-based play activities and sessions, (2) a music play resource kit, and (3) session planning and caregiver tip sheets (Table 2). At the start of sessions, the therapist and caregiver used a session planning sheet to identify child needs for that day (e.g., symptom management; movement; developmental), which provided focus for that specific session. This was followed by 30 min of music play with the caregiver and child. During session closure, using caregiver tip sheets, the music therapist would provide information about ways the caregiver and child could use music-based play to manage distress and maintain a sense of family normalcy while hospitalized and at home following discharge. The child and caregiver also received a music play resource kit to encourage independent use of music play strategies between scheduled sessions, and at home following discharge.

**TABLE 2:** Active music engagement (AME) intervention components and theoretical principles.

**Intervention component**	**Theoretical principles**
Component 1: music-based play activities	(1) Predictable environment provides a structure that supports child competence. Therapist uses familiar music activities to provide structure and increase child’s ability to predict what will happen in their environment. (2) Leveled activities help ensure success and support child competence. Therapist tailors physical activity requirements to meet the individual needs of each child. Enables child success and engagement during periods of high or fluctuating symptom distress. (3) Opportunities to make independent decisions support child autonomy. Children choose from a variety of music play activities, and each activity includes a wide range of materials. Activities include a wide range of materials and activity options so that the child can make choices for self and others. Therapist uses improvisational techniques to follow child-initiated changes in their music making (e.g., child changes tempo or style of playing). (4) Activities structured to support caregiver–child interaction. Activities structure and support reciprocal caregiver–child interactions. The therapist individualizes experiences to support increased frequency and quality of interactions.
Component 2: music play resource kit	Supports independent use of music play to manage distress between therapist-led sessions. Activities mirror content from therapist-led sessions. The kit includes: (1) Professional CD recording of music composed and/or arranged specifically for the AME intervention. (2) Age-appropriate musical instrument and play materials that correspond to each activity. (3) Activity cards designed to give children/caregivers at-a-glance information on ways they can use their kit.
Component 3: session planning and tip sheets	(1) Promotes caregiver competence about how children use play to cope and ways to engage their child in music play during the transplant period. (2) Promotes caregiver autonomy by empowering caregivers with skills/resources to support their child during treatment. (3) Supports caregiver–child relationships through normalizing music-based play activities.

*Reprinted from Russ et al. (2020).*

All study sessions were delivered by the same MT-BC who was trained on study procedures and the AME protocol before the study opened to enrollment. All sessions were audio-recorded and the MT-BC completed quality assurance checklists following each session to ensure consistent delivery and adherence to the intervention protocol.

#### Data Collection

Caregivers completed self- and proxy-report measures of caregiver and child symptom distress up to 30 days prior to their child’s HSCT admission (baseline), before and after AME sessions 2, 4, and 6, and in a clinic visit 100 days post-transplant (follow-up). Caregivers also completed semi-structured interviews within 2 weeks of follow-up. All measures and the semi-structured interviews were administered by trained staff who were not involved in the delivery of AME and interviews were audio-recorded to facilitate their transcription.

**FIGURE 2:**
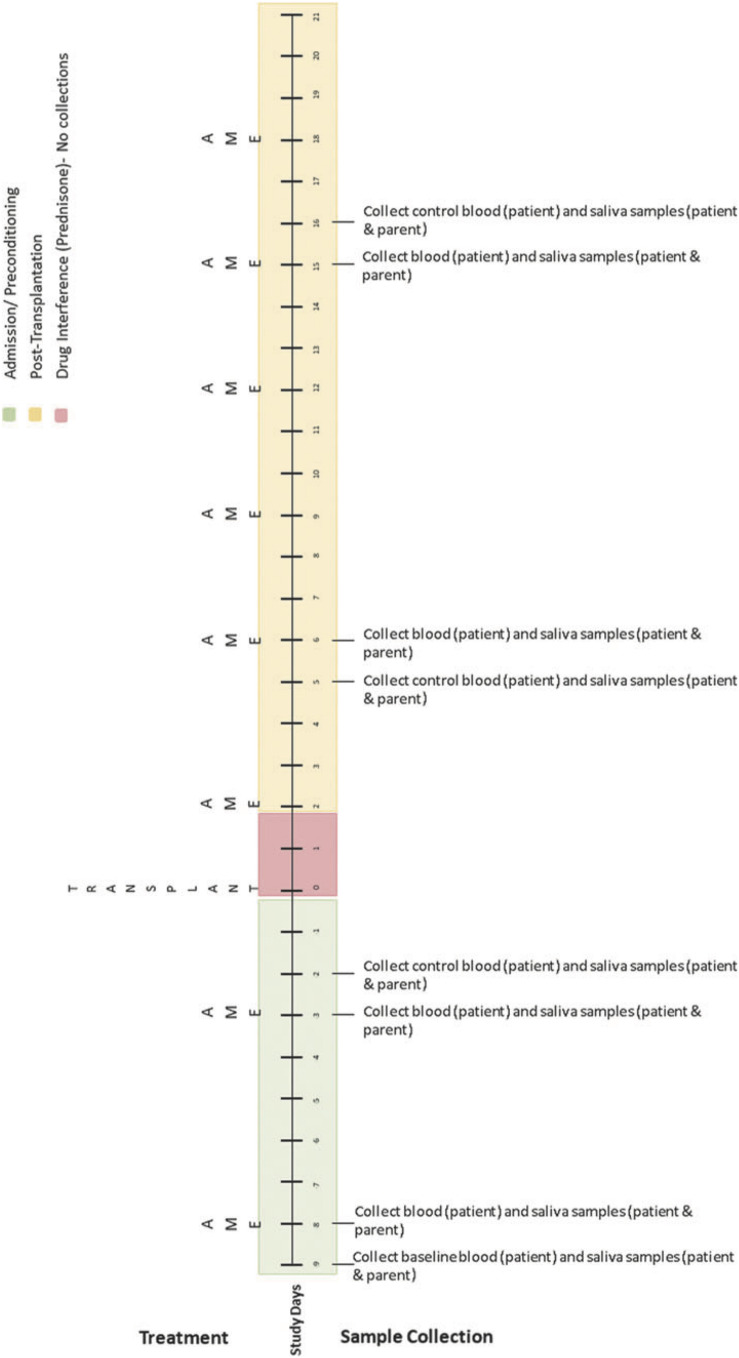
Collection schedule for children undergoing autologous transplant. Reprinted from Russ et al. (2020).

**FIGURE 3:**
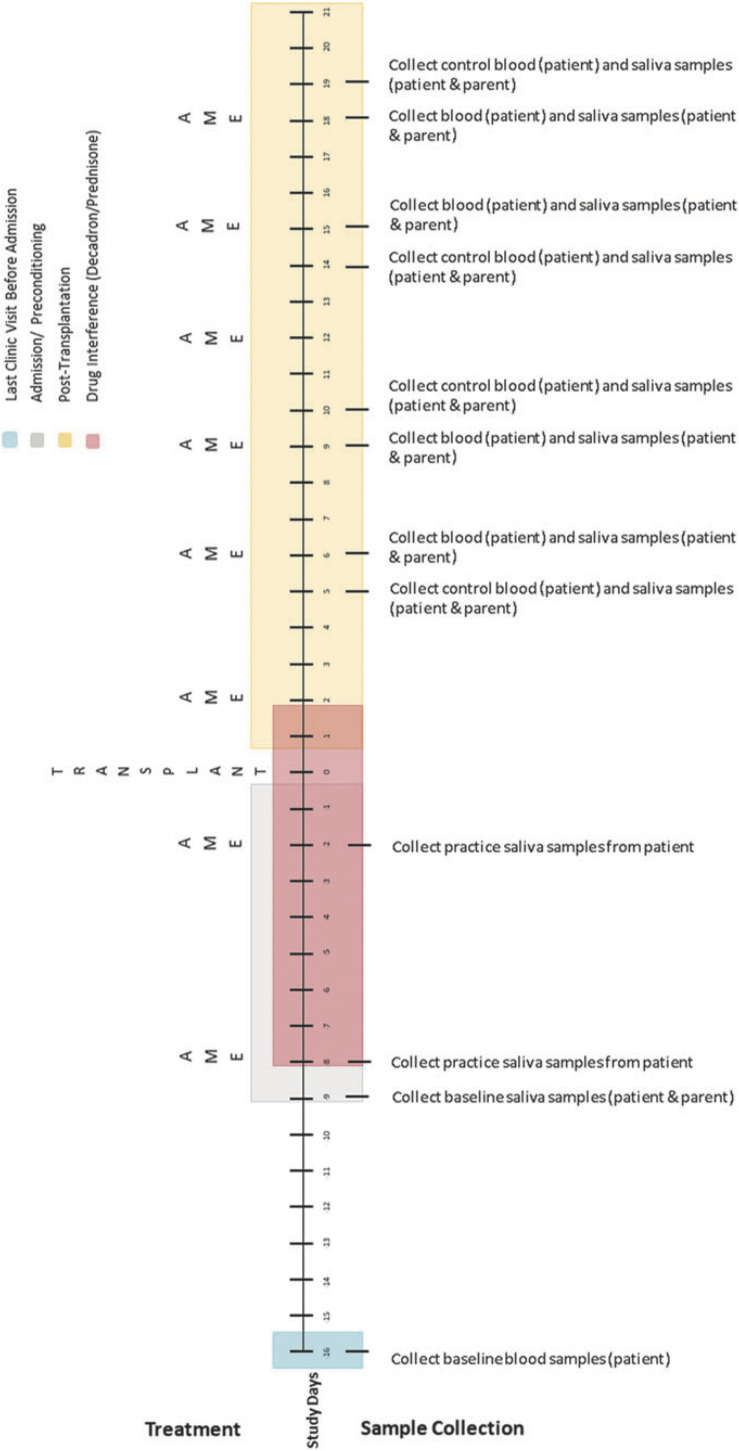
Collection schedule for children undergoing allogenic transplant. Reprinted from Russ et al. (2020).

**FIGURE 4:**
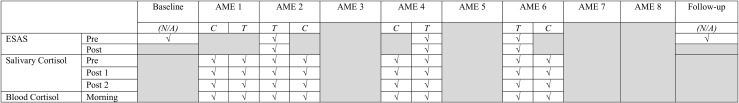
Simplified collection schedule for self- and proxy-report measures (ESAS), salivary cortisol, and blood cortisol. As shown in Figures 2, 3, the correspondence of AME sessions and data collection varied for children undergoing autologous and allogenic transplants; for illustrative purposes, this figure depicts days for a child undergoing an autologous transplant. Pre, Post, and Morning refer to the time of day during which samples were collected. Pre indicates that data collection occurred before the AME session, whereas Post indicates collection occurred after the session (Post 1 and Post 2) are used to indicate that data were collected twice after the session. C and T refer to control and treatment condition days within each pair of days on which data were collected for a given AME session.

Biological data collection featured sampling of saliva (from caregivers and children) and blood (from children only). Saliva samples were collected up to 1 h before AME sessions, up to 1 h following, and again 1 to 3 h later using the passive drool method (i.e., caregivers and children drooled into a collection tube; see Törnhage, 2009). At the time of collection, participants provided information about whether they had recently eaten or had a drink and when they last slept. Blood was collected via a draw from children’s central line as part of their daily blood collection (between 4 and 7 a.m.). These data were collected using the same schedule on four treatment days (on which AME sessions occurred) and four control days (on which AME sessions did not occur) over the course of HSCT such that treatment and control days were always 1 day apart. Collection schedules differed for children undergoing autologous and allogenic stem cell transplants. See Figures 2–4 for additional details.

### Measures

#### Self- and Proxy-Report Measures

Caregivers completed eight items from the Edmonton Symptom Assessment System (ESAS; Bruera et al., 1991; Richardson and Jones, 2009; Hui and Bruera, 2017) eight times over the course of the study: at baseline, before and after AME sessions 2, 4, and 6, and at follow-up. The ESAS includes nine-items that measure symptom burden including physical and emotional distress. ESAS uses an 11-point numeric rating scale (0 = no symptoms; 10 = worst possible). Caregivers were asked to rate their child’s and their own distress using the timeframe “at this moment.” Caregivers completed five items about their child’s symptom distress (depression, anxiety, pain, tiredness, and nausea) and three items about their own distress (depression, anxiety, and tiredness). Composite indices of child and caregiver distress were calculated as the sum of the items regarding each topic.

#### Semi-Structured Interviews

Interviews began with an open-ended question to capture the overall experience (i.e., “Please tell me about your experience of participating in the music play sessions with your child”); followed by specific questions about perceived benefit (or non-benefit) of AME in managing stress, experiences with saliva collection, and the value of different intervention components (i.e., tip sheets, goal setting worksheet, and kit use).

#### Cortisol

Saliva samples were stored on dry ice immediately following collection and then frozen at −80°C. Samples were then assayed using an R&D Systems cortisol parameter assay. Following collection, blood samples were sent immediately to a pathology laboratory, where they were assayed using a Beckman Coulter UniCel DxI800 immunoassay system. The minimum cortisol concentration that can be detected using this assay is 0.40 μg/dL.

### Data Analysis

Data analysis was conducted in two phases that reflected the primary and secondary aims of the study. *A priori* thresholds for feasibility were based on prior AME studies (see Robb et al., 2008, 2017) and reflect recruitment rates and usable data that would be necessary in future trials. Given that the number of children (age 3–8 years) who receive HSCT is relatively low, we established higher thresholds (>75%) for enrollment, session completion, and data collection. In the first phase, the percentage of eligible child/caregiver dyads that consented to participate in the study was calculated and compared to a pre-determined feasibility threshold of 75%. To address our first research question, the percentage of AME sessions that participating families completed was also calculated and compared to an acceptability threshold of 75%. The reasons that eligible dyads declined participation and planned intervention activities that did not occur as scheduled were also reported.

To address our second research question we calculated the percentage of planned self- and proxy-report measures that were collected and compared these percentages to a feasibility threshold of 75%. We also calculated the percentage of planned saliva and blood-based cortisol samples that were collected and the subset of these that yielded useable data. The percentage of cortisol samples collected were compared to feasibility thresholds for caregivers (85%) and children (75%). The percentage of samples collected that yielded useable data were compared to thresholds for caregivers (75%) and children (60%). Feasibility thresholds were set lower for child cortisol samples given greater possibility for challenges due to treatment side effects.

To address research questions three and four, semi-structured interviews were transcribed and then subject to a deductive content analysis^54^ using MaxQDA (version 18.2). Interviews were coded for categories and concepts and agreement was obtained by consensus. Question four was also addressed by graphing all responses on the self- and proxy-report ESAS measures of distress to visualize trends^55^ and then calculating Tau-U statistics (Parker et al., 2011) to compare reported distress prior to AME sessions 2, 4, and 6 with distress following those sessions. In this study negative values of Tau-U indicate that levels of distress decreased from pre- to post-session.

The fifth and final research question was addressed in a similar fashion, but using blood and salivary cortisol levels. All data were graphed and then Tau-U statistics were computed to compare all unique pairs of cortisol data points between the treatment and control conditions. For blood cortisol levels, the Tau-U statistics were based on the data collected at the beginning of each day, and the contrasts of interest were between these morning cortisol levels of treatment versus control days. Negative values of Tau-U indicate that levels of cortisol in blood were lower on treatment condition days than on control days. For the salivary cortisol levels, the Tau-U statistics were based on the area under the curve with respect to increase (AUC_I_) on treatment and control days, given that the collection schedule (featuring three samples on each day) allowed for values of AUC_I_ to be calculated. The AUC_I_ corresponds to the change in cortisol from the initial measurement (Pruessner et al., 2003), and therefore comparing values of the AUC_I_ on treatment days to control days allows us to examine whether the amount of cortisol produced during and following the AME session (on treatment days) differed from the amount produced at approximately the same times on control days, when AME did not occur. Negative values of Tau-U for comparisons of the AUC_I_ indicate that salivary cortisol levels were lower relative to baseline on treatment condition days than on control days.

Prior to the calculation of the Tau-U statistics, data were inspected for evidence of serial conditioning effects by comparing all unique pairs of data points *within* the control condition, which would be indicated by a systematic (i.e., non-random) trend in data points collected over the course of days corresponding to control condition. When these effects were detected, the Tau-U statistics were adjusted accordingly. The adjusted or unadjusted Tau-U statistics were then used to estimate *z* scores that corresponded to a standardized effect size of the treatment on cortisol levels for each participant and the sample as a whole.^56^

## Results

### Phase One Analyses: Primary Aims

#### Study Participation and Completion

During a 4-month enrollment period, four caregiver-child dyads were eligible to participate in the study. Of these, four (or 100%) agreed to participate; this was 25% higher than our feasibility threshold of 75%. One dyad (dyad 3) completed all eight AME sessions as scheduled; two dyads completed seven sessions as scheduled. In one case (dyad 2) the child was discharged from the hospital before the last session occurred, and in the other (dyad 4) one session had to be rescheduled due to caregiver illness. In one case (dyad 1) three sessions had to be rescheduled because the child refused the session on the day it was to occur. Therefore, across participants 27 of 32 sessions (84.4%) occurred as scheduled, which exceeded the 75% feasibility threshold. However, it is important to note two points: first, with the exception of the final session for dyad 2 (prior to which the child was discharged), all dyads completed eight sessions after rescheduling; and second, that the primary caregiver (the child’s mother) was not able to be present for five of these sessions in the case of dyad 1 and for two sessions in the cases of dyads 3 and 4. In these cases the child’s father or grandparent was present.

#### Data Collection

Caregivers were scheduled to complete the self- and proxy-report measures of distress (the ESAS items) eight times over the course of the study (at baseline, before and after AME sessions 2, 4, and 6, and at follow-up). Although adults other than the child’s mother provided data when the mother was unable to do so, only ESAS data provided by the mother was used to avoid introducing variability due to different reporters into the analyses. The mother provided data at four time points for dyads 1 and 2, and six time points for dyads 3 and 4. Therefore, across all four caregivers the measure was complete 20 out of 32 times, for a completion rate of 62.5%, which fell below the feasibility threshold for the sample overall; however, the feasibility threshold was reached for dyads 3 and 4.

Saliva samples were collected from caregivers and children. As noted above, three samples were scheduled to be collected from both participants on each of the eight collection days. Therefore, 24 samples were to be collected from each caregiver and child. Across the four caregivers, 69 (71.9%) of 96 possible samples were collected, all of which were viable, meaning that cortisol concentrations could be calculated from the samples. Three of four caregivers provided 18 or more samples, all of which were viable (≥75%). Two children (child 1 and child 4) provided saliva for 54.2 and 20.8% of samples, respectively; 84.5% of these samples were viable for child 1 and all were viable for child 4. The remaining two children refused saliva sampling.

Blood samples were collected on all eight collection days for all children, exceeding the feasibility threshold of 75%. Across children, 27 of 32 samples were viable (84.4%), exceeding the feasibility threshold of 75%. All samples that were not viable were collected from the same child (child 2), and yielded cortisol concentrations that fell outside the bounds of the assay.

#### Caregivers’ Perspectives on Data Collection

Deductive content analysis of semi-structured interviews yielded the categories and sub-categories displayed in Table 3 along with corresponding exemplar quotations. Overall, caregivers perceived the saliva collection protocol as stressful for themselves and their child. While caregivers expressed a clear desire to provide and obtain saliva samples for the study, they also found the process to be challenging. All of the children experienced difficulty providing saliva samples using the passive drool technique. And, although the actual collection procedure was not difficult for parents, the frequency of collections and the need to refrain from eating and drinking for a prolonged period created further disruptions in their day, such as finding time to eat and shower. Given challenges with saliva collection, some caregivers shared that if possible, they would have preferred use of urine, blood, or stool.

**TABLE 3:** Categories, subcategories, and exemplar quotations for semi-structured interviews.

**Category/subcategory**	**Exemplar quotations**
Category: challenges of saliva collection during HSCT	So I think that in retrospect, that piece of getting, at least a child that young, I don’t know what other, I know you did blood work as well, maybe taking the blood work a little more frequently, as opposed to the spit, at least for that age, (it) was a little bit much. (parent 4)
Subcategory: parent collection stressful for parents	The saliva collection was a bit much. You know what, I’m going to be honest with you, I would have rather given blood. I would have rather been like here, go ahead and take some blood and let’s get this done. (parent 4) As far as like doing the study, I would go a really long time without eating. And so when I finally had a chance to eat and we weren’t busy, I ate and I felt really guilty that I was messing up the results of this study possibly. (parent 2)
Subcategory: child collection stressful for parents and children	I don’t know how viable it is to get a 3-year-old or a child that young to spit. That was pretty challenging. I did feel a little, and this is just me, a little stressed about getting (child) to spit, getting him to actually spit. (parent 4) she did not at all want to do this spit in the cup thing. Tried really hard and like stressed her out… (parent 2)
Category: AME intervention as beneficial during HSCT	(the questionnaire asked) Has your kid smiled? And I’m like, oh no, not for weeks. And I didn’t even notice. It was like, oh she’s just tired and she’s here. But then when we would do music, she would kind of get excited and smile a little bit and get excited and kind of giggle. And that was really, really good to see. My first couple of times, like I started crying cause I was like, oh, she’s happy. That was really cool. (parent 2)
Subcategory: helpful to set goals	(referencing session planning sheet) Today he just needs to be a kid, because every day he needs to be a kid. (parent 4)
Subcategory: mitigating emotional distress	… (when) she was kind of stressed out, she would sing in her wagon That was huge. That’s something she had not done before. it was like, she kinda knew, I don’t know, like instinctively knew like singing calmed her down. So that was a really a surprise seeing her self soothe herself with music. (parent 2) It take the stress off, definitely trying to calm him down cause it helps calm him when he wasn’t feeling so good. (parent 3)
Subcategory: mitigating physical distress	She didn’t sleep for two like 2 or 3 days straight. She could not sleep because she was in so much pain. And then [Music Therapist] came in and did music and she fell asleep. So that was, that was pretty magical. (parent 2)
Subcategory: kit use between sessions	So we’ve been… kind of getting him up moving. We did the frog and the alligator more so that he would dance as opposed to doing the books. (parent 3)
Subcategory: using tip sheets	I think they were helpful information just in general. I mean it definitely helped the different ways to see how it the different activities would help. And what activities help with which issue. (parent 3)

### Phase Two Analyses: Secondary Aims

#### Caregivers’ Perceptions of AME Benefits

Based on the analysis of transcribed semi-structured interviews, the AME intervention, adapted to the longer stay associated with HSCT, was a positive and beneficial experience (Table 3). Caregivers noted marked improvement in their child’s emotional and physical distress both during and after AME sessions. Caregivers reported that the opportunity to witness their child become happy and playful (when they had previously been withdrawn, in pain, and/or experiencing nausea) eased their own distress, and provided ways to connect with and support their child. In addition, session planning sheets, parent tip sheets, and the music resource kit functioned as intended – parents shared that these resources raised awareness and provided skills and resources to successfully manage their child’s distress outside of therapist-led sessions. Not only did parents find ways to use music, but they also witnessed their child using music to self-manage their own distress.

**FIGURE 5:**
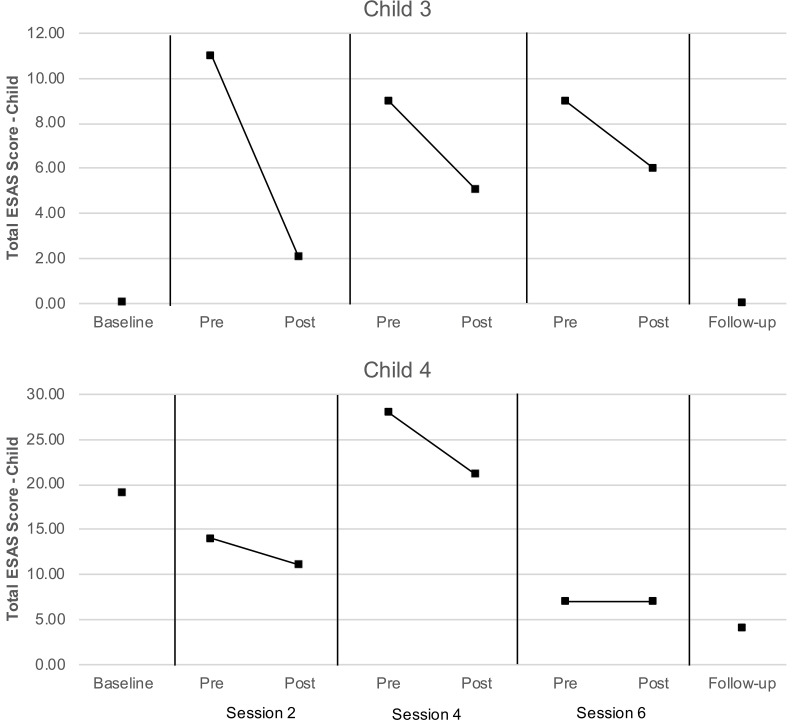
Child ESAS scores (as reported by caregivers).

The proportion of data available for the proxy-report ESAS measures regarding child distress exceeded the feasibility threshold for dyads 3 and 4, and therefore these data were subject to analyses as described above. As can be seen in Figure 5, total symptom distress scores reported by caregivers for their children were lower at post-session than at pre-session for both children at each assessment. Values of Tau-U were calculated for the two children with data exceeding the feasibility threshold (child 3 and 4) to compare pre- and post-session distress scores. Levels of child distress reported for child 3 were lower at post-session than at pre-session at a level that was nearly significant (*T* = −1.00, *SD* = 0.51, *z* = −1.96, *p* = 0.050); a similar pattern was not observed for child 4 (*T* = −0.22, *SD* = 0.51, *z* = −0.44, *p* = 0.663). No serial conditioning effects were detected in pre-session levels of distress child 3 (*T* = −0.67, *SD* = 0.64, *z* = −1.04, *p* = 0.296) or child 4 (*T* = −0.33, *SD* = 0.64, *z* = −0.52, *p* = 0.602); therefore, no corrections for such effects were required.

#### Differences in HPA-Axis Activity

**FIGURE 6:**
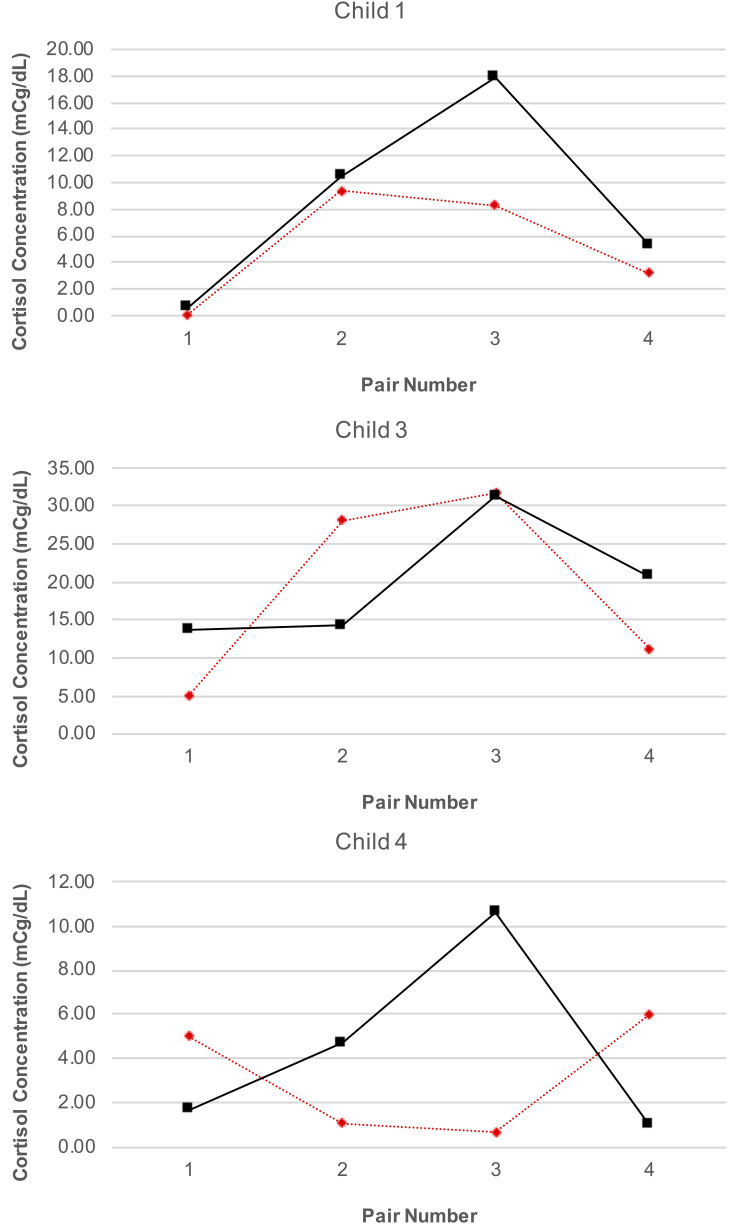
Child blood cortisol concentrations.

Our analyses were confined to the viable blood samples collected from all four children and the saliva samples collected from three caregivers. Table 4 reports the blood cortisol concentrations for child, while Figure 6 provides a visualization of the data for the three children for whom the proportion of viable samples exceeded the feasibility threshold. An inspection of this figure suggests two patterns in the data: first, regardless of condition (treatment or control), cortisol concentrations increased over time among child 1 and child 3, peaking at time of the third session, and then declining by the time of the fourth session. A similar pattern was observed for child 4, but only for days corresponding to the treatment condition. Second, there was no consistent difference in cortisol levels between conditions within pairs of days. The latter pattern was confirmed by the results of the analyses using the Tau-U statistic, in which no contrasts were significant for any subject (Child 1: *T* = 0.38, *SD* = 0.43, *z* = 0.87, *p* = 0.866; Child 3: *T* = 0.13, *SD* = 0.43, *z* = 0.29, *p* = 0.773; Child 4: *T* = 0.13, *SD* = 0.43, *z* = 0.29, *p* = 0.773) or for the group of subjects as a whole (*T* = 0.21, *z* = 0.83, *p* = 0.405). Note that no serial conditioning effects were detected in cortisol levels across control condition days for any child (Child 1: *T* = 0, *SD* = 0.49, *z* = 0, *p* = 1; Child 3: *T* = 0.33, *SD* = 0.49, *z* = 0.68, *p* = 0.497; Child 4: *T* = 0, *SD* = 0.49, *z* = 0, *p* = 1), and therefore no corrections for such effects were required.

**TABLE 4:** Blood cortisol concentrations.

** Subject**	** Collection pair**	** Salivary cortisol concentration (μg/dL)**	
		
		** Control condition**	** Treatment condition**
1	1	0.50	0.60
	2	9.40	10.50
	3	8.30	17.90
	4	3.20	5.30
2	1	- - -^1^	- - -^1^
	2	- - -^1^	- - -^1^
	3	22.90	9.90
	4	0.40	- - -^1^
3	1	5.10	13.70
	2	28.10	14.30
	3	31.70	31.30
	4	11.20	20.80
4	1	5.00	1.70
	2	1.10	4.70
	3	0.70	10.60
	4	6.00	1.00

*^1^Cortisol concentration was below the minimum detectable range of the assay (0.40 μg/dL).*

**FIGURE 7:**
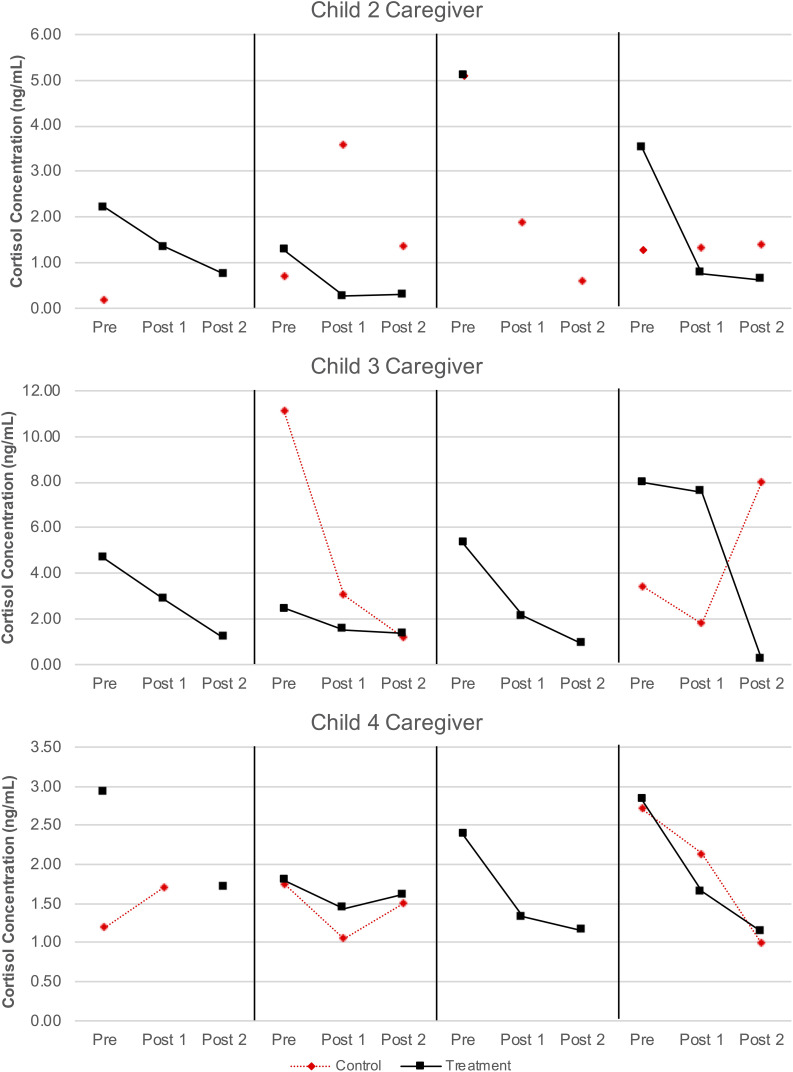
Caregiver salivary cortisol concentrations.

The caregivers of children 2, 3, and 4 provided viable cortisol samples for at least 18 (75%) of the 24 data collection points. Salivary cortisol concentrations for each viable sample are reported in Table 5 and visualized in Figure 7. Figure 7 also displays the linear trend (depicted by a line of best fit) in cortisol levels for every day on which at least two cortisol samples were collected (which comprised all but one treatment condition day across the three caregivers who provided salivary cortisol samples and eight control condition days). As can be seen in the figure, on each treatment condition day the nature of the linear trend was toward lower levels of cortisol after the first, pre-AME sample was taken, as indicated by the negative slope for the line of best fit. In contrast, cortisol decreased after the first sample only on three control condition days.

**TABLE 5:** Salivary cortisol concentrations and areas under the curve.

**Subject**	**Collection pair**	**Condition**	** Salivary cortisol concentration (ng/mL)**			
			
			**Pre-AME**	**Post- AME 1**	**Post- AME 2**	**AUC_I_**
2	1	Control	0.20	—	—	—
		Treatment	2.20	1.33	0.74	−1.60
	2	Control	0.69	3.58	1.37	3.23
		Treatment	1.29	0.27	0.30	−1.52
	3	Control	5.11	1.88	0.61	−5.48
		Treatment	5.10	—	—	—
	4	Control	1.28	1.32	1.41	0.11
		Treatment	3.52	0.77	0.63	−4.20
3	1	Control	—	—	—	—
		Treatment	4.65	2.85	1.17	−3.54
	2	Control	11.13	3.06	1.22	−13.03
		Treatment	2.47	1.54	1.37	−1.48
	3	Control	—	—	—	—
		Treatment	5.36	2.13	0.92	−5.45
	4	Control	3.43	1.81	7.99	0.66
		Treatment	7.99	7.57	0.25	−4.29
4	1	Control	1.20	1.71	—	—
		Treatment	2.92	—	1.70	—
	2	Control	1.75	1.05	1.50	−0.83
		Treatment	1.80	1.44	1.61	−0.46
	3	Control	—	—	—	—
		Treatment	2.39	1.33	1.15	−1.68
	4	Control	2.71	2.14	0.99	−1.43
		Treatment	2.83	1.65	1.14	−2.03

The areas under the curve with respect to increase (AUC_I_) were calculated as described above and are reported in Table 5. We then used these AUC_I_ values to calculate Tau-U statistics. For the caregiver of child 2, these analyses revealed that the AUC_I_ was smaller on treatment condition days than control condition days at the trend level (*T* = −1.00, *SD* = 0.65, *z* = −1.55, *p* = 0.121). Similar differences were not observed for the caregivers of Child 3 or 4 (for both, *T* = 0, *SD* = 0.65, *z* = 0, *p* = 1). As in the case for blood samples, no serial conditioning effects were detected across control condition days (for all children, *T* = −1.00, *SD* = 1.00, *z* = −1.00, *p* = 0.317).

## Discussion

The current study was designed to address two sets of aims. The first set of aims concerned the feasibility/acceptability of implementing the AME intervention during HSCT and collecting data from young children (age 3 to 8 years) in this clinical context, and the first question among these aims was whether a sufficient proportion of eligible caregiver-child dyads would consent to participate in the study. The fact that over a 4-month period 100% of eligible caregiver-child dyads agreed to participate in our study (exceeding our feasibility threshold by 25%) strongly suggests that although HSCT is a high-risk treatment characterized by uncertainty and high symptom distress, caregivers of young children (age 3.51 to 4.53) are willing to enroll and provide biological samples making it possible to recruit a sample of sufficient size for a SCD study. Moreover, the fact that nearly 85% of AME sessions occurred as scheduled indicates that it is possible to implement the intervention while children are undergoing this treatment.

Consent rates and session completion rates for this pilot study are consistent with prior AME trials. In one prior pilot study, we had 80% enrollment and 100% session completion rate (Robb et al., 2017). In our current multi-site trial looking at AME during a 3-day inpatient admission we had 79% enrollment and 90% session completion, even at one of the participating hospitals where music therapy was a new service. Recruitment and informed consent were completed by a study member who was not part of the clinical care team; however, the music therapist had been working on the unit full-time for 5 years. The opportunity to establish good working relationships with bedside staff and the availability of music therapy as a support service commonly seen on the unit may have contributed to high session completion rates. In addition, although the majority of participants were new to the music therapist, she had worked with two families at some point prior to their transplant and enrollment in the study. Finally, due to the nature of the intervention, presentation of the AME study is often met with a high level of interest from children and their caregivers.

Having established that participants can be recruited and the intervention can be delivered, the next question was whether data could be collected according to the study design. On this point, our results were mixed. In the case of two dyads, caregivers completed the self- and proxy-report measures of distress at a rate that met our feasibility threshold. Where cortisol data collection was concerned, each child provided all eight blood samples, and for three children all samples were viable. Collecting saliva samples from children proved difficult, such that only two children provided any samples and in both cases the proportion of samples provided fell well below the feasibility threshold. In contrast, caregivers provided 72% of the scheduled saliva samples, which approached the feasibility threshold of 75%. The difference in the proportion of samples provided by children and their caregivers may be explained, in part, by the side effects of HSCT, which include dry mouth and oral sores that can discourage children from providing samples. In addition, high symptom distress coupled with younger child age may have contributed to lower sample acquisition. Although our enrollment criteria included children age 3–8 years, the average age for enrolled participants was 3.84 years making it impossible to examine whether older children would have experienced difficulty providing samples.

The results of the semi-structured interviews corroborate this explanation for low rates of collection among children, and also offer additional reasons that caregivers did not provide saliva samples at every time point. Chief among these was the fact the number and frequency of samples required by the SCD interfered with caregivers’ schedules, preventing them from drinking, eating, or showering over a number of hours. This, together with the need to collect samples from the same caregiver on each collection day across the 8 weeks of their participation and that these data were being collected in the context of a highly distressing treatment regimen (cf., Best et al., 2001; Virtue et al., 2014) had a compounded effect that resulted in additional burden for children and their caregivers.

The secondary aims of the study were to assess the benefits of AME via caregiver interviews, child ESAS scores, and cortisol levels in children and their caregivers. Interview data indicate the AME intervention was beneficial in reducing child emotional and physical distress, both during and after therapist-led sessions. In addition, caregivers experienced relief in seeing these improvements in their child and reported that they and their child used music-play activities and resources outside session to manage distress. These findings suggest that AME offers not only immediate relief during therapist-led sessions, but that it also helped children and their caregivers learn to use new strategies to manage their own distress between sessions.

In the case of the two children (child 3 and 4) whose caregivers provided sufficient ESAS data, visual inspection and analyses of child distress scores converged with those obtained from the semi-structured interviews. In both cases, the child’s caregiver consistently reported lower levels of child distress after the AME occurred, relative to before. Interestingly, Child 3, who had the most consistent (and significant) reduction in symptom distress had one of the hardest courses of treatment – meaning the child had an allogeniec transplant (which is associated with more frequent and intense side effects, compared with autologous transplants), and this child had Graft Versus Host Disease (GVHD) that was Stage 4, Grade 3. GVHD is unique to HSCT because it is essentially the immune system that is being transplanted. This makes it possible for both the child’s existing immune system to attack the new stem cells (graft failure) and the newly transplanted immune system to attack the child’s body (GVHD). Typical manifestation of GVHD includes skin rash, liver dysfunction and gastrointestinal symptoms like pain, nausea and diarrhea (Nassereddine et al., 2017). Future studies that take into account diagnosis and treatment intensity may help determine for whom AME is most beneficial.

Child 4 also had an allogeneic transplant, but for SCD. This is important because children with SCD have typically not yet encountered chemotherapy. While SCD is a disease with high symptom burden already, chemotherapy and prolonged hospitalization are difficult to prepare a family for. This often leads to higher levels of uncertainty for both the child and caregiver, and a potentially harder time adjusting to the experience of these new, intense side-effects (Hulbert and Shenoy, 2018). This is apparent in higher distress scores at baseline (compared with Child 3), and may explain markedly higher scores mid-treatment when symptom distress is particularly difficult.

Our analyses of cortisol concentrations in blood samples for the three children who provided samples revealed a consistent pattern of cortisol over time, with cortisol levels increasing from the first pair of data collection days to peak at the third pair, before declining by the fourth pair (with the caveat that for one child this pattern was observed only on treatment condition days). This pattern is consistent with other studies examining distress during HSCT, where distress increases over time as side-effects from and ambiguity about treatment outcomes increase, and then has a gradual decline toward the end of treatment as severity of symptoms decreases and counts stabilize (Phipps et al., 2002; Vrijmoet-Wiersma et al., 2009). There were also no differences in levels of blood cortisol concentrations on treatment and control condition days, though this must be interpreted with caution due to the schedule of the SCD. It is possible that on control condition days falling the day after a treatment day levels of morning cortisol are lower due to the AME session that occurred the day before; similarly, higher levels of morning cortisol on treatment days may reflect the fact that AME did not occur on the previous day and had yet to occur on that day.

Our analysis of salivary cortisol samples provided by the three caregivers who met our feasibility threshold revealed that caregivers were more likely to exhibit decreases in cortisol on days they participated in an AME than on days when they did not. This suggests that AME may have some impact on caregivers’ patterns of diurnal HPA-axis activity, or at least the portion of that activity which coincided with the times of day when samples were collected. To the extent that this activity reflects caregivers’ perceived stress, the AME intervention may have the potential to lower caregivers’ stress levels, a possibility that is congruent with the results obtained from semi-structured interviews conducted with caregivers. However, it must also be noted that only one caregiver was found to have systematically lower aggregate or overall levels of cortisol on treatment days relative to control days, and that this difference was observed at the trend level. It may therefore be the case that while AME has the potential to change the slope of caregivers’ diurnal cortisol, that change is not large enough to result in systematic differences in the overall level of cortisol produced.

### Limitations and Future Directions

Our results clearly indicate that it is feasible to implement the AME intervention in the context of HCST. The results about collecting data with sufficient frequency to execute a SCD were mixed, as were the results about the benefits of AME that this design yielded. The mixed results regarding the benefits of AME clearly call for additional research. The question is what form that research should take. A randomized control trial (RCT) offers the same protections against threats to internal validity provided by a SCD, and is therefore an appealing option. The problem is that an RCT investigation into the benefits of AME would be very likely to be under-powered due to the low incidence rate of pediatric patients (age 3–8 years) undergoing use of HCST (Transplant Activity Report, 2019).

The question then becomes how to modify the procedures for a SCD study to minimize both the burden that data collection places on families and the amount of missing data. One clear lesson from the current study is that collecting salivary cortisol samples from younger children (age 3–4) in this treatment setting is infeasible. Another is that interpreting a single, waking blood cortisol sample is problematic. Taking one additional blood sample after the AME session occurred (and at the same times on control-condition days) would allow for a comparison of diurnal activity across conditions, which could be more readily interpreted. The caveat is that collecting additional blood samples can elevate the risk of infection, and so this additional sample would ideally coincide with blood draws for usual monitoring during transplant and must be taken in consideration with the appropriate weight-based thresholds in daily blood draws for younger patients (cf., Peplow et al., 2019).

Similar adjustments to collecting salivary cortisol samples from caregivers are warranted. Some caregivers disliked the passive drool method of saliva collections describing the process as “gross.” Switching to oral swabs is therefore recommended. It is also important to allow more flexibility in the collection schedule with caregivers. The HPA axis is a sluggish neurophysiological system in which change occurs gradually. Some parents delayed self-care activities, such as taking a shower, in anticipation of a scheduled collection. As such, reassuring parents that these activities will not interfere with collection is important. Allowing a caregiver to take a shower before providing a sample is acceptable, assuming the shower does not last for more than approximately 20 min. Similarly, allowing a parent to eat or drink in between samples is fine, as long as they do not do so immediately before providing a sample and try to avoid certain items that will contaminate the sample (e.g., highly-acidic beverages, such as orange juice).

It may be worthwhile to consider using additional or alternative stress biomarkers for use with children and/or caregivers. Collecting other biomarkers that are sequestered in saliva will pose precisely the same challenges as collecting salivary cortisol, and while it may be possible to collect blood samples from caregivers, the frequency with which those samples would have to be collected to support a SCD may be problematic. However, like the HPA axis, the autonomic nervous system (ANS) is highly responsive to stress, and there are many non-invasive measures of ANS activity that could serve as a stress biomarker. These include measures of global ANS activity, such as blood pressure, as well as more specific measures of the ANS’ two branches, the parasympathetic and sympathetic nervous systems, both of which can be indexed via cardiac monitoring.

Finally, as demonstrated in this pilot study, despite our focus on understanding the biological effects of active music on child and caregiver stress it is important to include brief symptom distress measures that are sensitive to change and to conduct participant interviews. As with any measure there are limitations; therefore, it is essential that investigators use diverse forms of measurement and inquiry to fully capture changes in child and caregiver distress.

## Conclusion

This study investigated whether cortisol could be used as a stress biomarker in a SCD study to explore the effects of AME on children undergoing HSCT, as well as their caregivers. Our results suggest that it is possible to recruit a sufficient number of families to participate in such a study, and that the AME can be implemented as designed. However, they also suggest that collecting salivary cortisol from children and caregivers with the frequency required for a SCD creates additional burden for families already undergoing a difficult treatment. Nevertheless, we found that families are eager to participate in the study of an intervention they value and provide biological samples which underscores the need for devising more innovative, less burdensome approaches for understanding the neurophysiological impacts of AME in future research.

## Data Availability Statement

The de-identified data supporting the conclusions of this article can be obtained from the corresponding author via a written data-sharing agreement.

## Ethics Statement

This study, which involved human participants, was reviewed and approved by Indiana University Institutional Review Board. Written informed consent to participate in this study was provided by the participating parent (legal guardian) for self and for their child.

## Author Contributions

AH and KR were responsible for data entry and management. KR and SH were responsible for analysis of biological specimens, while KS and SR analyzed qualitative data. CK was responsible for delivery AME sessions. SH and SP oversaw all other analyses. SH and SR prepared the initial draft of the manuscript, to which all authors subsequently made contributions. All authors contributed to the study design.

## Conflict of Interest

The authors declare that the research was conducted in the absence of any commercial or financial relationships that could be construed as a potential conflict of interest. The reviewer KM declared a past collaboration with one of the authors SH to the handling editor.

## References

[B1] Barrera M. D’Agostino N. M. Gibson J. Gilbert T. Weksberg R. Mlkin D. (2004). Predictors and mediators of psychological adjustment in mothers of children newly diagnosed with cancer. *Psychooncology* 13 630–641. 10.1002/pon.765 15334531

[B2] Best M. Streisand R. Catania L. Kazak A. E. (2001). Parental distress during pediatric leukemia and posttraumatic stress symptoms (PTSS) after treatment ends. *J. Pediatr. Psychol.* 26 299–307. 10.1093/jpepsy/26.5.299 11390572

[B3] Bruce M. (2006). A systematic and conceptual review of posttraumatic stress in childhood cancer survivors and their parents. *Clin. Psychol. Rev.* 26 233–256. 10.1016/j.cpr.2005.10.002 16412542

[B4] Bruera E. Kuehn N. Miller M. J. Selmser P. Macmillan K. (1991). The edmonton symptom assessment system (ESAS): a simple method for the assessment of palliative care patients. *J. Palliat. Care* 7 6–9. 10.1177/082585979100700202 1714502

[B5] Costanzo E. S. Sood A. K. Lutgendorf S. K. (2011). Biobehavioral influences on cancer progression. *Immunol. Allergy Clin. N. Am.* 31 109–132. 10.1016/j.iac.2010.09.001 PMC3011980 21094927

[B6] Glaser R. Kiecolt-Glaser J. K. (2005). Stress-induced immune dysfunction: implications for health. *Nat. Rev. Immunol.* 5 243–251. 10.1038/nri1571 15738954

[B7] Graf A. Bergstraesser E. Landolt M. A. (2013). Posttraumatic stress in infants and preschoolers with cancer. *Psychooncology* 22 1543–1548. 10.1002/pon.3164 22911462

[B8] Guilcher G. M. T. Truong T. H. Saraf S. L. Joseph J. J. Rondelli D. Hsieh M. M. (2018). Curative therapies: allogeneic hematopoietic cell transplantation from matched related donors using myeloablative, reduced intensity, and nonmyeloablative conditioning in sickle cell disease. *Semin. Hematol.* 55 87–93. 10.1053/j.seminhematol.2018.04.011 29958564 PMC6130892

[B9] Gunnar M. Quevedo K. (2007). The neurobiology of stress and development. *Annu. Rev. Psychol.* 58 145–173. 10.1146/annurev.psych.58.110405.085605 16903808

[B10] Hatfield E. Cacioppo J. T. Rapson R. L. (1993). Emotional contagion. *Curr. Dir. Psychol. Sci.* 2 96–99. 10.1111/1467-8721.ep10770953

[B11] Hobbie W. L. Stuber M. Meeske K. Wissler K. Rourke M. T. Ruccione K. (2000). Symptoms of posttraumatic stress in young adult survivors of childhood cancer. *J. Clin. Oncol.* 18 4060–4066. 10.1200/JCO.2000.18.24.4060 11118467

[B12] Hui D. Bruera E. (2017). The edmonton symptom assessment system 25 years later: past, present, and future developments. *J. Pain Sympt. Manag.* 53 630–643. 10.1016/j.jpainsymman.2016.10.370 PMC5337174 28042071

[B13] Hulbert M. Shenoy S. (2018). Hematopoietic stem cell transplantation for sickle cell disease: progress and challenges. *Pediatr. Blood Cancer* 65:e2726 10.1002/pbc.27263 29797658

[B14] Ingerski L. M. Shaw K. Gray W. N. Janicke D. M. (2010). A pilot study comparing traumatic stress symptoms by child and parent report across pediatric chronic illness groups. *J. Dev. Behav. Pediatr.* 31 713–719. 10.1097/DBP.0b013e3181f17c52 20814337 PMC2975762

[B15] Kang D. H. Rice M. Park N. J. Turner-Henson A. Downs C. (2010). Stress and inflammation: a biobehavioral approach for nursing research. *Western J. Nurs. Res.* 32 730–760. 10.1177/0193945909356556 20624936

[B16] Kangas M. Henry J. L. Bryant R. A. (2002). Posttraumatic stress disorder following cancer. a conceptual and empirical review. *Clin. Psychol. Rev.* 22 499–524. 10.1016/s0272-7358(01)00118-0 12094509

[B17] Kazak A. E. Barakat L. P. (1997). Brief report: parenting stress and quality of life during treatment for childhood leukemia predicts child and parent adjustment after treatment ends. *J. Pediatr. Psycol.* 22 749–758. 10.1093/jpepsy/22.5.749 9383934

[B18] Kazak A. E. Barakat L. P. Meeske K. Christakis D. Meadows A. T. Casey R. (1997). Posttraumatic stress, family functioning, and social support in survivors of childhood leukemia and their mothers and fathers. *J. Consult. Clin. Psychol.* 65 120–129. 10.1037/0022-006X.65.1.120 9103741

[B19] Kazak A. E. Baxt C. (2007). Families of infants and young children with cancer: a post-traumatic stress framework. *Pediatr. Blood Cancer* 49 (Suppl.), 1109–1113. 10.1002/pbc.21345 17943959

[B20] Kazak A. E. Boeving C. A. Alderfer M. A. Hwang W. T. Reilly A. (2005). Posttraumatic stress symptoms during treatment in parents of children with cancer. *J. Clin. Oncol.* 23 7405–7410. 10.1200/jco.2005.09.110 16157936

[B21] Kazak A. E. Cant M. C. Jensen M. M. McSherry M. Rourke M. T. Hwang W. (2003). Identifying psychosocial risk indicative of subsequent resource use in families of newly diagnosed pediatric oncology patients. *J. Cliical Oncol.* 21 3220–3225. 10.1200/JCO.2003.12.156 12947055

[B22] Kazak A. E. Stuber M. L. Barakat L. P. Meeske K. Guthrie D. Meadows A. T. (1998). Predicting posttraumatic stress symptoms in mothers and fathers of survivors of childhood cancers. *J. Ame. Acad. Child Adoles. Psychiatry* 37 823–831. 10.1097/00004583-199808000-00012 9695444

[B23] Knafl K. A. Deatrick J. A. (2002). The challenge of normalization for families of children with chronic conditions. *Pediatr. Nurs.* 28:49.

[B24] Lane L. (1991). The Effect of a Single Music Therapy Session on Hospitalized Children as Measured by Salivary Immunoglobulin A, Speech Pause Time, and a Patient Opinion Likert Scale. (Electronic Thesis or Dissertation). Available at: https://etd.ohiolink.edu/ (accessed October 18, 2019).

[B25] Langeveld N. E. Grootenhuis M. A. Voute P. A. de Haan R. J. (2004). Posttraumatic stress symptoms in adult survivors of childhood cancer. *Pediatr. Blood Cancer* 42 604–610. 10.1002/pbc.20024 15127415

[B26] Moore I. M. (2004). Advancing biobehavioral research in childhood cancer. *J. Pediatr. Oncol. Nurs.* 21 128–131. 10.1177/1043454204264400 15296039

[B27] Nassereddine S. Rafei H. Elbahesh E. Tabbara I. (2017). Acute graft versus host disease: a comprehensive review. *Anticancer Res.* 37 1547–1555. 10.21873/anticanres.11483 28373413

[B28] Padgett D. A. Glaser R. (2003). How stress influences the immune response. *Trends Immunol.* 24 444–448. 10.1016/s1471-4906(03)00173-x 12909458

[B29] Parker R. I. Vannest K. J. Davis J. L. Sauber S. B. (2011). Combining non-overlap and trend for single-case research: tau-u. *Behav. Ther.* 42 284–299. 10.1177/0145445511399147 21496513

[B30] Peplow C. Assfalg R. Beyerlein A. Hasford J. Bonifacio E. Ziegler A. G. (2019). Blood draws up to 3% of blood volume in clinical trials are safe in children. *Acta Paediatr.* 108 940–944. 10.1111/apa.14607 30291644 PMC6587985

[B31] Phipps S. Dunavant M. Garvie P. A. Lensing S. Rai S. N. (2002). Acute health-related quality of life in children undergoing stem cell transplant: I. Descriptive outcomes. *Bone Marrow Trans.* 29 425–434. 10.1038/sj.bmt.1703377 11919733

[B32] Post-White J. Fitzgerald M. Savik K. Hooke M. C. Hannahan A. B. Sencer S. F. (2009). Massage therapy for children with cancer. *J. Pediatr. Oncol. Nurs.* 26 16–28. 10.1177/1043454208323295 19074355

[B33] Pruessner J. C. Kirschbaum C. Meinlschmid G. Hellhammer D. H. (2003). Two formulas for computation of the area under the curve represent measures of total hormone concentration versus time-dependent change. *Psychoneuroendocrinology* 28 916–931. 10.1016/s0306-4530(02)00108-7 12892658

[B34] Rensen N. Gemke R. J. van Dalen E. C. Rotteveel J. Kaspers G. J. (2017). Hypothalamic-pituitary-adrenal (HPA) axis suppression after treatment with glucocorticoid therapy for childhood acute lymphoblastic leukaemia. *Cochrane Database Syst. Rev.* 11:CD008727 10.1002/14651858.CD008727.pub4 PMC6486149 29106702

[B35] Richardson L. A. Jones G. W. (2009). A review of the reliability and validity of the edmonton symptom assessment system. *Curr. Oncol.* 16:55.2644623 10.3747/co.v16i1.261 PMC2644623 19229371

[B36] Robb S. L. (2000). The effect of therapeutic music interventions on the behavior of hospitalized children in isolation: developing a contextual support model of music therapy. *J. Music Ther.* 37 118–146. 10.1093/jmt/37.2.118 10932125

[B37] Robb S. L. (2003a). “Coping and chronic illness: music therapy for children and adolescents with cancer,” in *Music Therapy in Pediatric Healthcare*, ed. Robb S. R. (Silver Spring, MD: American Music Therapy Association).

[B38] Robb S. L. (2003b). Designing music therapy interventions for hospitalized children and adolescents using a contextual support model of music therapy. *Music Ther. Perspect.* 21 27–40. 10.1093/mtp/21.1.27

[B39] Robb S. L. Clair A. A. Watanabe M. Monahan P. O. Azzouz F. Stouffer J. W. (2008). A non-randomized [corrected] controlled trial of the active music engagement (AME) intervention on children with cancer. *Psychooncology* 17 699–708. 10.1002/pon.1301 18033724

[B40] Robb S. L. Haase J. E. Perkins S. M. Haut P. R. Henley A. K. Knafl K. A. (2017). Pilot randomized trial of active music engagement intervention parent delivery for young children with cancer. *J. Pediatr. Psychol.* 42 208–219. 10.1093/jpepsy/jsw050 27289068 PMC5896608

[B41] Robb S. L. Hanson-Abromeit D. (2014). A review of supportive care interventions to manage distress in young children with cancer and parents. *Cancer Nurs.* 37 E1–E26. 10.1097/NCC.0000000000000095 24936752

[B42] Robinson K. E. Gerhardt C. A. Vannatta K. Noll R. B. (2007). Parent and family factors associated with child adjustment to pediatric cancer. *J. Pediatr. Psychol.* 32 400–410. 10.1093/jpepsy/jsl038 17085460

[B43] Russ K. A. Holochwost S. J. Perkins S. M. Stegenga K. Jacob S. A. Delgado D. (2020). Cortisol as an acute stress biomarker in young hematopoietic stem cell transplant patients/caregivers: active music engagement protocol. *J. Alteran. Comp. Med.* 26 424–434. 10.1089/acm.2019.0413 PMC7232696 32073877

[B44] Santacroce S. (2002). Uncertainty, anxiety, and symptoms of posttraumatic stress in parents of children recently diagnosed with cancer. *J. Pediatr. Oncol. Nurs.* 19 104–111. 10.1177/104345420201900305 12066262

[B45] Steele R. G. Long A. Reddy K. A. Luhr M. Phipps S. (2003). Changes in maternal distress and child-rearing strategies across treatment for pediatric cancer. *J. Pediatr. Psychol.* 28 447–452. 10.1093/jpepsy/jsg035 12968036

[B46] Stevens B. Croxford R. McKeever P. Yamada J. Booth M. Daub S. (2006). Hospital and home chemotherapy for children with leukemia: a randomized cross-over study. *Pediatr. Blood Cancer* 47 285–292. 10.1002/pbc.20598 16200556

[B47] Stuber M. L. Christakis D. A. Houskamp B. Kazak A. E. (1996). Posttrauma symptoms in childhood leukemia survivors and their parents. *Psychosomatics* 37 254–261. 10.1016/s0033-3182(96)71564-5 8849502

[B48] Stuber M. L. Kazak A. E. Meeske K. Barakat L. Guthrie D. Garnier H. (1997). Predictors of posttraumatic stress symptoms in childhood cancer survivors. *Pediatrics* 100 958–964. 10.1542/peds.100.6.958 9374564

[B49] Törnhage C. (2009). Salivary cortisol for assessment of hypothalamic-pituitary-adrenal axis function. *Neuroimmunomodulation* 16 284–289. 10.1159/000216186 19571589

[B50] Transplant Activity Report (2019). *Number of Transplants per Year.* Available at: https://bloodstemcell.hrsa.gov/sites/default/files/bloodstemcell/data/transplant-activity/transplants-year-age-group.pdf (accessed October 18, 2019).

[B51] Virtue S. M. Manne S. L. Mee L. Bartell A. Sands S. Gajda T. M. (2014). Psychological distress and psychiatric diagnoses among primary caregivers of children undergoing hematopoietic stem cell transplant: an examination of prevalence, correlates, and racial/ethnic differences. *Gen. Hosp. Psychiatry* 36 620–626. 10.1016/j.genhosppsych.2014.08.010 25246347 PMC4329245

[B52] Vrijmoet-Wiersma C. M. J. Egeler R. M. Koopman H. M. Lindahl Norberg A. Grootenhuis M. A. (2009). Parental stress before, during, and after pediatric stem cell transplantation: a review article. *Support. Care Cancer* 17 1435–1443. 10.1007/s00520-009-0685-4 19572154 PMC2775902

[B53] Walco G. A. Conte P. M. Labay L. E. Engel R. Zeltzer L. K. (2005). Procedural distress in children with cancer: self-report, behavioral observations, and physiological parameters. *Clin. J. Pain* 21 484–490. 10.1097/01.ajp.0000146166.15529.8b 16215333

